# A Phylogenetic Study of the ANT Family Points to a preANT Gene as the Ancestor of Basal and euANT Transcription Factors in Land Plants

**DOI:** 10.3389/fpls.2019.00017

**Published:** 2019-01-29

**Authors:** Melissa Dipp-Álvarez, Alfredo Cruz-Ramírez

**Affiliations:** Molecular and Developmental Complexity Group, Unidad de Genómica Avanzada, Laboratorio Nacional de Genómica para la Biodiversidad, Centro de Investigación y de Estudios Avanzados del IPN, Guanajuato, Mexico

**Keywords:** AP2-like, euANT, basalANT, phylogeny, *cis-*elements

## Abstract

Comparative genomics has revealed that members of early divergent lineages of land plants share a set of highly conserved transcription factors (TFs) with flowering plants. While gene copy numbers have expanded through time, it has been predicted that diversification, co-option, and reassembly of gene regulatory networks implicated in development are directly related to morphological innovations that led to more complex land plant bodies. Examples of key networks have been deeply studied in *Arabidopsis thaliana*, such as those involving the AINTEGUMENTA (ANT) gene family that encodes AP2-type TFs. These TFs play significant roles in plant development such as the maintenance of stem cell niches, the correct development of the embryo and the formation of lateral organs, as well as fatty acid metabolism. Previously, it has been hypothesized that the common ancestor of mosses and vascular plants encoded two ANT genes that later diversified in seed plants. However, algae and bryophyte sequences have been underrepresented from such phylogenetic analyses. To understand the evolution of ANT in a complete manner, we performed phylogenetic analyses of ANT protein sequences of representative species from across the Streptophyta clade, including algae, liverworts, and hornworts, previously unrepresented. Moreover, protein domain architecture, selection analyses, and regulatory *cis* elements prediction, allowed us to propose a scenario of how the evolution of ANT genes occurred. In this study we show that a duplication of a preANT-like gene in the ancestor of embryophytes may have given rise to the land plant-exclusive basalANT and euANT lineages. We hypothesize that the absence of euANT-type and basalANT-type sequences in algae, and its presence in extant land plant species, suggests that the divergence of pre-ANT into basal and eu-ANT clades in embryophytes may have influenced the conquest of land by plants, as ANT TFs play important roles in tolerance to desiccation and the establishment, maintenance, and development of complex multicellular structures which either became more complex or appeared in land plants.

## Introduction

### Colonization of Land by Plants

The plant terrestrialization process, also known as the colonization of land by plants, took place 425–490 million years ago (Sanderson, 2003). It has been hypothesized that such process occurred in a microbial moist biofilm as substrate, which could have been composed of fungi, bacteria, cyanobacteria, and green algae (Wellman and Strother, 2015). Prior to land colonization, freshwater green algae related to extant Charophyte algae had already evolved different types of forms; from unicellular organisms to multicellular filaments and thallose forms (Harrison, 2017). It is hypothesized that the first land plants originated from one of these types of ancestral algae from the order Zygnematales that were tolerant to desiccation and adapted to aerial conditions, which allowed them to survive in the previously mentioned substrate (Wellman and Strother, 2015; Ishizaki, 2017; Puttick et al., 2018). In turn, plant terrestrialization was critical for the formation of earth’s biosphere, a fundamental change that allowed the colonization of terrestrial environments by animals.

Several innovations allowed early plants to diversify and thrive on changing terrestrial environments, such as the transition from a gametophyte-dominant to sporophyte-dominant life form, the three-dimensional growth form, originated by changes in the division plane orientations of stem cells, the development of a sporophytic apical meristem, and the origin of new organs and tissues such as vasculature, roots, leaves, flowers, and seeds (Pires and Dolan, 2012; Ishizaki, 2017). It has been predicted that such innovations in the land plant body were the product of diversification, co-option, and reassembly of gene regulatory networks implicated in development; given that members of early diverging lineages of land plants share a set of highly conserved transcription factors (TFs) with flowering plants (Pires and Dolan, 2012; Bennett et al., 2014). In line with this idea, a comparative study carried out by Catarino et al. (2016), revealed that the number of TF families has barely increased in land plants after terrestrialization compared to the increase since the divergence of chlorophytes and streptophytes.

From an evolutionary perspective it has been proposed that the AINTEGUMENTA (ANT) family of TFs is conserved as part of the ancestral molecular toolkit that enabled life and development of plants on earth (Kim et al., 2006; Shigyo et al., 2006; Floyd and Bowman, 2007). Previously, it was hypothesized that the common ancestor of mosses and vascular plants encoded two ANT genes that later diversified in seed plants (Floyd and Bowman, 2007). However, algae and bryophyte sequences were underrepresented or lacking from previous phylogenetic analyses (Kim et al., 2006; Shigyo et al., 2006; Floyd and Bowman, 2007) and it is not clear if the origin of the basalANT and euANT subclades predated land plant divergence.

### ANT Lineage of Transcription Factors

*ANT* genes belong to the APETALA 2/ETHYLENE RESPONSE FACTOR (AP2/ERF) family of TFs characterized for containing the AP2/ERF DNA binding domain, which were first discovered in the *A. thaliana* AP2 protein, a critical protein for the establishment of the floral meristem (Kim et al., 2006). ANT proteins contain two AP2 domains separated by a linker region (25 amino acids) so they fit under the AP2-like subfamily of proteins (Riechmann and Meyerowitz, 1998; Supplementary Figure 1). The AP2-like subfamily is further divided in two different lineages: the euAP2 lineage, which mRNA has a miR172 target sequence in the post-domain region, and the AINTEGUMENTA (ANT) lineage, characterized by a 10- amino acid insertion in the first AP2 domain (R1) and a 1- amino acid insertion in the second AP2 domain (R2), see Supplementary Figure 1. In turn, the ANT lineage is divided into the basalANT and the euANT sublineages (Horstman et al., 2014). The main difference among eu and basalANT is that euANT proteins are defined by a long pre-domain region and four conserved motifs. The first motif, euANT1, consists of conserved amino acids (NSC[K/R][K/R]EGQ[T/S]R) in the 10-amino acid insertion located in the first AP2 domain. The other three motifs, euANT2 (WLGFSLS), euANT3 (PKLEDFLG) and euANT4 (TFGQR), are located in the pre-domain region of the euANT proteins as described in Supplementary Figure 1 (Kim et al., 2006).

*Arabidopsis thaliana* euANT TFs have been deeply studied for over 20 years. They are represented in a gene family that include eight members: *AINTEGUMENTA (ANT), AINTEGUMENTA-LIKE 1 (AIL1), BABY BOOM (BBM)*, and *PLETHORAs* (*PLT1, PLT2, PLT3, PLT5, PLT7*). They are considered master regulators of diverse developmental processes and are mainly expressed in dividing tissues where they regulate the formation and maintenance of stem cell niches and the correct development of the embryo, the root and shoot organs. The characterization of the *plt1*;*plt2* double mutant revealed that *PLT1* and *PLT2* are essential for proper development of *A. thaliana* roots. Both genes are expressed, in an auxin-dependent manner, at the basal part of the octant stage embryo and in the quiescent center in late embryogenesis, and are essential for the correct specification and development of both the embryonic and post-embryonic root meristems (Aida et al., 2004). On the other hand, overexpression (OE) of *BBM* (*PLT4*) or *PLT5* induces ectopic embryos on the aerial part of the seedling, while *PLT1* and *PLT2* OE induces ectopic root stem cell niches (reviewed in Horstman et al., 2014).

*Arabidopsis thaliana* PLT1, PLT2, and PLT3 are expressed in the root forming a gradient distribution with a maximum in the stem cell niche, this is also true for *Oryza sativa PLT* genes (Galinha et al., 2007; Li and Xue, 2011). The gradient distribution of these PLT proteins indicates that they act as a dose-dependent morphogen, because high levels of PLTs promote stem cell identity, while lower levels allow stem cell daughters to partially differentiate and keep mitotic activity (Galinha et al., 2007). However, when cells are displaced to the root region with a drastic lower concentration of PLTs, they lose proliferation capacity and terminally differentiate (Galinha et al., 2007). Seedlings of the homozygous triple mutant *plt1;plt2;plt3* developed by Galinha et al. (2007) show a fully differentiated embryonic root pole at 3 days post-germination (d.p.g.) and they never develop lateral roots. All together, these phenotypes position these specific euANT genes as master regulators of root development. Also, Hofhuis et al. (2013) showed that *PLT3, PLT5*, and *PLT7* are expressed in lateral root primordia, regulating lateral root development and preventing that lateral root primordia are formed close to one another. This study reveals that the genetic mechanism that regulates rhizotaxis and involves *PLT3, PLT5*, and *PLT7* is the same that controls phylotaxis in the *A. thaliana* shoot (Prasad et al., 2011).

In the case of *A. thaliana ANT*, it has been demonstrated that ANT TF plays a key role in flower development (Krizek, 1999). OE of *ANT* under the cauliflower mosaic virus 35S constitutive promoter (*35S::ANT*) causes the development of larger floral organs resulting from an increase in cell number in sepals. While larger petals, stamens, and carpels observed in ANT OE plants are the result of an increase in cell size.

*Arabidopsis thaliana* genes *WRINKLED1 (WRI1), WRINKLED3 (WRI2), WRINKLED4 (WRI4)*, and *ADAP* are members of the basalANT lineage. *WRI1* regulates fatty acid metabolism in the seed, binding to the *cis* region of glycolytic genes and its activity is localized mainly to the maturing embryo (To et al., 2012). WRI3 and WRI4 expression is localized to vegetative tissue and flowers where they activate cuticular wax production (Park et al., 2016) and *AthADAP* is necessary for Abscisic acid response and regulates drought response (Lee et al., 2009).

### ANT Transcription Factors in Bryophytes

Two basalANT-type TFs, homologs of the rice *SMALL ORGAN SIZE1* (*SMOS1*) gene, were found in *Physcomitrella patens*: *PpSMOS1-like1 and PpSMOS1-like2.* In rice, *SMOS1* acts downstream of auxins to regulate cell expansion during organ size control and the mutant phenotype, which has an overall smaller size of organs, was partially complemented by *PpSMOS1-like1* (Aya et al., 2014). On the other hand, there are four euANT genes encoded in the *P. patens* genome, *APB1-4*. They act as a molecular switch for the development of different types of stem cells and their expression is induced by auxin, similarly to what has been shown for *A. thaliana AIL* genes (Aoyama et al., 2012). No reports exist yet for ANT genes in Streptophyte algae and other bryophytes, aside from *Physcomitrella patens*.

### ANT Transcription Factors in Non-flowering Vascular Plants

In the C-fern *Ceratopteris richardii*, Bui et al. (2017) identified an ortholog of the *A. thaliana ANT* gene, *CrANT*, which has an expression pattern similar to *A. thaliana BBM* (in embryo development). The ectopic expression of *CrANT* in *C. richardii* gametophytes originated the spontaneous production of apogamous sporophytes that develop sporophyte-only vascular tissue, tracheids, and stomata. This suggests that *CrANT* has an important role in sexual reproduction and that *AthBBM* might have evolved from an ancestral *AIL* gene such as *CrANT*. A single euANT gene has been reported for both *Gnetum parvifolium* and *Pinus taeda* (gymnosperms) and their expression pattern in young primordia indicate that they might have an important role in lateral organ size control as well as in the development of the ovule (Shigyo and Ito, 2004; Yamada et al., 2008), very similar to what has been reported for AthANT (Klucher et al., 1996; Mizukami and Fischer, 2000).

So far evolutionary analyses that include ANT genes from Streptophyte algae and other bryophytes, apart from *P. patens*, have not been reported. Therefore, the main objective of this study was to explore in a more extensive and deep manner the evolutionary relationships among basalANT, euANT and, the first time described, preANT genes. In order to generate this more complete scenario on ANT gene evolution along the plant phylogeny, we set the following objectives: (i) to analyze and compare the structures and motif conservation in preANT, basalANT and euANT proteins; (ii) to explore how natural selection has influenced ANT gene evolution, (iii) to define differences between *cis*-regulatory elements in the preANT, basalANT, and euANT promoter regions, and (iv) to explore the evolutionary relationships of these genes along the plant kingdom.

## Methods

### Phylogenetic Analysis of ANT Proteins

To reconstruct the phylogeny of the ANT lineage of TFs among the Streptophyta clade, we conducted a search for basalANT and euANT homologs by querying the coding sequence of eight *A. thaliana* euANT genes (*ANT, AIL1, PLT1, PLT2, AIL6, BBM, PLT5*, and *PLT7*) and three *A. thaliana* basalANT genes (*WRI1, ADAP*, and *WRI4*) against Phytozome^1^ (Goodstein et al., 2012), the Conifer Genome Integrative Explorer^2^ (Nystedt et al., 2013), the OneKP^3^ database, the *Klebsormidium nitens* genome project^4^, and the recently published genomes from *Azolla filiculoides* and *Salvinia cucullata* (Li et al., 2018). We then selected the *A. thaliana* AP2 gene and homologous sequences from other seed plants, as outgroups to explore the relationships among ANT genes.

Putative ANT TF coding sequences from selected angiosperms, gymnosperms, ferns, lycophytes, bryophytes, charophytes, and a chlorophyte, were obtained and translated selecting the longest open reading frame. Our final database included a total of 114 ANT protein sequences from 23 different species as well as 10 outgroup sequences (taxa, sequence length, and ID shown on Supplementary Table 1). AA sequences were aligned with the MAFFT version 7 online service (Katoh et al., 2017) using the L-INS-i method that incorporates local pairwise alignment information (Katoh et al., 2005). The alignment was manually edited using Jalview 2.10.3 (Waterhouse et al., 2009), deleting uninformative gaps and regions of the alignment that were poorly aligned. The final alignment consists of 124 taxa and contains 1854 alignment positions of which 1748 are phylogenetically informative (Supplementary Data Sheet 1). We then identified the best partitioning scheme and the best-fit evolution model for each section of the protein alignment (pre-domain, AP2-R1, linker, AP2-R2, and post-domain regions) with PartitionFinder 2.1.1 run on the CIPRES Science Gateway (Miller et al., 2011; Lanfear et al., 2017) with the greedy search algorithm (Lanfear et al., 2012), the RAxML option (Stamatakis, 2014), and the corrected Akaike Information Criterion (AICc) to compare models of molecular evolution. The selected best-fitting models of AA substitution were VT+G+F, for the pre- and post-domain regions; JTTDCMUT+I+G, for the AP2-R1 domain; LG4M+G, for the linker; and JTT+G for the AP2-R2 domain. A Maximum Likelihood (ML) phylogenetic reconstruction was then performed with the whole alignment matrix in RAxML version 8.2.10 (Stamatakis, 2014) on CIPRES with the four distinct models identified by PartitionFinder 2.1.1, the GAMMA model of rate heterogeneity and with 1000 rapid bootstrap inferences to evaluate the analysis. The best tree with bootstrap branch labels was then edited using the iTOL online software (Letunic and Bork, 2016).

### ANT Protein Structure Analysis

To characterize the protein structure of ANT genes among Streptophyta, we analyzed the position of the reported ANT-type AP2-R1 and AP2-R2 domains (Riechmann and Meyerowitz, 1998; Kim et al., 2006) in the 99 AA sequences retrieved. The presence of the euANT motifs reported by Kim et al. (2006), as well as new conserved motifs and their AA composition was also assessed using the Multiple Em for Motif Elicitation (MEME) version 4.12.0 online software^5^ (Bailey and Elkan, 1994), run to search for 40 conserved motifs with a minimum and maximum length of 4 and 10 AAs, respectively.

### Selection Analysis for ANT Genes

**FIGURE 1:**
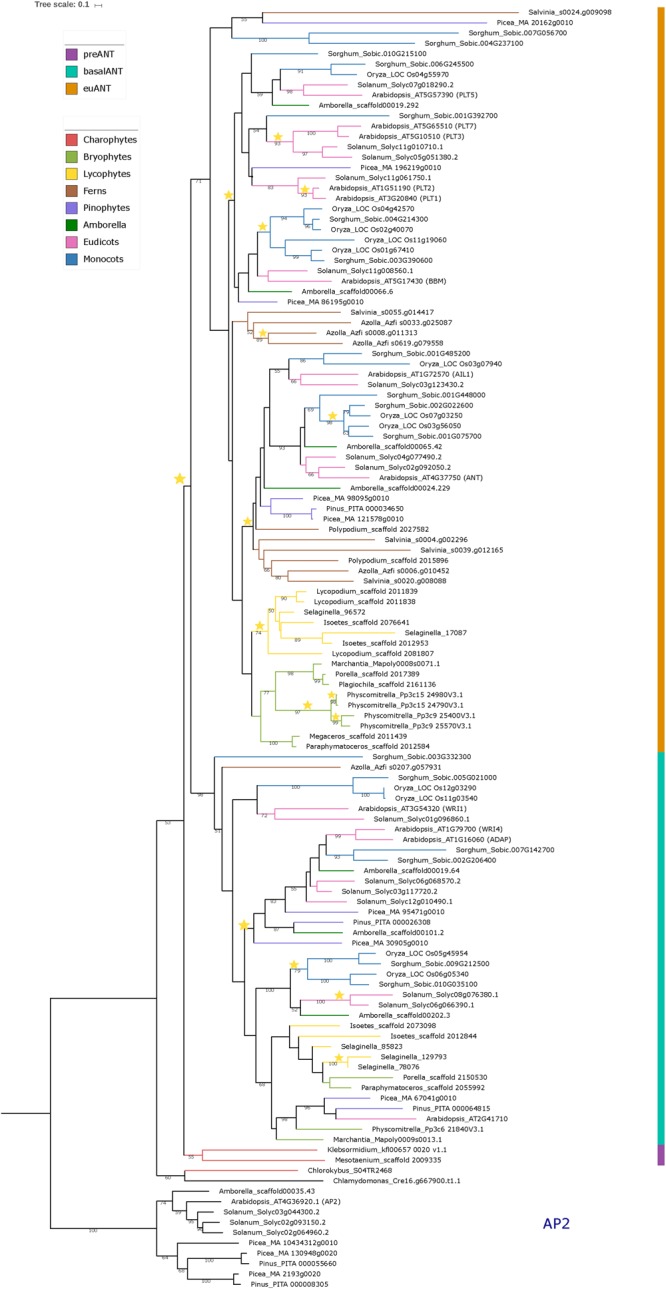
Phylogeny of the ANT gene clade across Streptophyta. Rooted Maximum Likelihood tree constructed on RAxML containing a total of 114 ANT protein sequences from 23 different taxa as well as 10 AP2 outgroup sequences. Bootstrap support was calculated using 1000 replicates. Only bootstrap values over 50% are shown on branches. Colored bar indicates ANT gene clades. Branch colors represent the taxonomic classification to which the taxa belong. Stars represent duplication events. See Supplementary Table 1 for species names.

To find signatures of natural selection along the protein coding sequence of euANT and basalANT genes we performed two types of Fixed Effects Likelihood (FEL) analyses (Kosakovsky Pond and Frost, 2005) and a Mixed Effects Model of Evolution (MEME) test (Murrell et al., 2012) with the HyPhy software package in the DataMonkey online server (Kosakovsky Pond and Frost, 2005). The input for both tests was a codon alignment of the protein coding region of all the ANT genes in this study (Supplementary Table 1) and the ML phylogenetic tree deduced with RAxML (see Phylogenetic analysis in Results) (Figure 1). The FEL approaches were designed to measure strength of selection, as well as compare positive and negative pervasive selection on sites in the basalANT clade (basalANT clade as foreground and the rest of the sequences as background) with that in the euANT clade (euANT clade as foreground and the rest of the sequences as background). For each of the FEL tests the ratio of non-synonymous to synonymous substitutions (*w* = dN/dS) was calculated for the foreground and background sets of sequences. Significance was determined for each codon position with a *p*-value < 0.05 for the test dN≠dS. The MEME approach, in which the *w* can vary through sites, was carried out to identify episodic diversifying selection affecting a proportion of branches (Murrell et al., 2012) and significance was assessed at *p* < 0.05.

### Putative *cis*-Regulatory Elements in ANT Genes

To identify putative *cis*-regulatory elements (CREs) in the promoter region of ANT genes, a 2.5 kb fragment upstream the ATG of ANT genes from *O. sativa, A. thaliana, A. trichopoda, P. patens, M. polymorpha*, and *Chlamydomonas reinhardtii* was extracted using Phytozome^6^. The 2.5 kb 5′ region flanking the *K*. *nitens* ANT gene (KFL_006570020, GenBank:DF237606.1; Hori et al., 2014) was obtained from the National Center for Biotechnology Information (NCBI)^7^ site. Sequences were then analyzed, using the PlantPAN 2.0 Multiple promoter analysis online tool (Chow et al., 2016), for consensus CREs. Results were filtered to keep the number and detailed position of MYB, WUSCHEL-related homeobox (WOX), ethylene response factor (ERF), auxin response factor (ARF), and citokynin response regulator (ARR-B) *cis-*elements found in each ANT gene promoter region.

## Results and Discussion

### Phylogeny of the ANT Lineage in Streptophyta

To better understand the origin and evolution of ANT genes we performed a Maximum Likelihood phylogenetic analysis based on the basalANT and euANT protein sequences we retrieved from representative species across the Streptophyta clade (Supplementary Table 1).

In Figure 1 we show the rooted phylogenetic tree for ANT, which shows that the single *C. reinhardtii* (unicellular Chlorophyte algae) and *Chlorokybus atmophyticus* (unicellular Streptophyte algae) sequences appear as a sister clade to a major clade composed of two clades (53% bootstrap support); the land plant ANT clade and a clade harboring the sequences from *Mesotaenium caldariorum* and *K. nitens. Chlamydomonas reinhardtii*, and *C. atmophyticus* sequences have an intra AP2-R1 AA insertion, but this sequence is distinct to that found in euANT proteins, the so called euANT1 motif. On the other hand, sequences from *M. caldariorum* and *K. nitens* already bear the euANT1 motif.

Since this is the first report in which a phylogeny for the ANT group includes Streptophyte algae ANT-like sequences, we hypothesize that *C. reinhardtii* and *C. atmophyticus* ANT-like sequences represent the putative ancestral sequence of the ANT group. Meanwhile, because of the position of the single *M. caldariorum* and *K. nitens* sequences and the presence of the euANT1 motif in the first AP2 domain in these genes, we decided to name these two sequences as preANT (Figure 1, marked with purple). Within the land plant ANT clade two highly supported clades are recovered; the basalANT (98%) (Figure 1, cyan bar) and euANT (71%) (Figure 1, orange bar) clades that were reported in previous phylogenies of ANT genes (Kim et al., 2006; Floyd and Bowman, 2007; Yamada et al., 2008).

The basalANT clade (Figure 1, cyan bar) has two subclades; a clade that groups the AthWRI1 sequence with its closely related sequences from *S. lycopersicum, O. sativa* (with two paralogs), and *Sorghum bicolor* (36%), and the other which consists of two subclades: the WRI4/ADAP clade that originated from a duplication event in the ancestor of seed plants (Figure 1, yellow star) and that clustered the AthWRI4 and AthADAP sequences together with sequences from *S. lycopersicum, S. bicolor, A. trichopoda, P. taeda*, and *Picea abies* sister to a clade of sequences that includes bryophyte, lycophyte, *Amborella trichopoda*, monocot and eudicot basalANT sequences which clustered with Arabidopsis_AT2G41710. All the sequences from the latter clade, share the characteristic of having incomplete AP2-R1 or AP2-R2 domains (see alignment in Supplementary Figure 2), which could be the result of different selective pressures driving the fixation of changes that lead to AA losses in the DNA-binding part of the protein in these taxa. Also, we detected duplication events within this clade in lycophytes, and angiosperms (Figure 1, yellow stars). WRI1 and WRI4/ADAP clades share a common ancestor with Azolla_Azfi_s0207.g057931, a fern sequence (51%). No other fern sequences were nested within the basalANT clade, which could be the result of incomplete sampling or loss of basalANT members.

Little is known about sequences belonging to the basalANT clade, aside from the role of *AthWRI1* in the regulation of fatty acid metabolism in the seed (To et al., 2012), of *AthWRI4* in cuticular wax biosynthesis (Park et al., 2016), and *AthADAP* a key TF for abscisic acid response (Lee et al., 2009). Recent experimental evidence points to the role of ABA in sex determination and transpiration in early land plants, millions of years before the ABA pathway was co-opted to modulate seed dormancy and water balance (McAdam et al., 2016). We can hypothesize that the ancient function of WRI/ADAP proteins could be related mainly to an ABA-dependent pathway that secured adaptation to a desiccated environment via modulating pore function for carbon dioxide and oxygen exchange and controlled water exchange, which requires the generation of an impermeable wax cover. This hypothesis could be tested with the functional characterization of basalANT genes from non-seed plants. We can also speculate that the duplication of WRI/ADAP genes in seed plants (Figure 1, yellow star) may have led to the acquisition of new expression patterns by positive selection to regulate fatty acid metabolism, dormancy, and water exchange in the seed and floral tissues.

Two major sister clades formed within the euANT (Figure 1, orange bar), one of them includes AthANT and AthAIL1 (48%). All the euANT sequences from bryophytes formed a subgroup within the ANT/AIL1 clade (43%). The sequences from Liverworts (*Porella pinnata, Plagiochila asplenoiides*, and *M. polymorpha*) clustered together (98%) as a sister clade to the clade containing the four *P. patens* sequences, forming the Setaphyta clade (Puttick et al., 2018). The contrast in number of euANT copies within the moss *P. patens* and the liverworts in this study has to do with the fact that the moss lineage has experienced events of Whole Genome Duplication (WGD) and retention of paralogs after these events (Figure 1, yellow stars) (Lang et al., 2018). The hornwort sequences (*Megaceros tosanus* and *P. hallii*) appeared as a sister clade (100%) to the Liverwort/Mosses group. The sequences from lycophytes (*Isoetes* sp., *Selaginella moellendorffii*, and *Lycopodium deuterodensum*) were grouped together in a clade (74%) sister to the bryophyte sequences. Floyd and Bowman (2007) also recovered the lycophyte sequences in their Bayesian analysis of euANT genes as sister clade to the ANT/AIL1 clade. All the sequences from ferns, were recovered in a sister clade to the bryophyte/lycophyte clade, although with low bootstrap support (38%). This clade also harbors sequences from pinophytes, *A. trichopoda*, eudicots and monocots closely related to AthANT and AthAIL1. A clade of just *A. filiculoides* and *S. cucullata* sequences was recovered as sister to the major ANT/AIL1 clade, the bootstrap support for this clade is low (<50) but it could have its origin after a duplication of the euANT gene in ferns. It would be interesting to see if this clade maintains its position when more fern genomes become available and a better sampling of ANT genes is possible.

The other euANT major clade in this phylogenetic estimation includes all the PLT/BBM sequences from *A. thaliana* and their closely related sequences from *P. abies, A. trichopoda, S. lycopersicum, O. sativa*, and *S. bicolor.* This clade was also resolved in previous euANT phylogenies (Shigyo and Ito, 2004; Kim et al., 2006; Floyd and Bowman, 2007; Yamada et al., 2008) but no pinophyte sequences were resolved within this clade before. In our phylogeny, Picea_MA_86195g0010 was included in the BBM clade, and Picea_MA_196219g0010 resolved within the PLT1/2 clade. This, together with the fact that no bryophyte, lycophyte or fern sequences were clustered within this clade, could mean that the PLT/BBM clade had its origins after an event of duplication in the common ancestor of seed plants (Figure 1, yellow star). Within the BBM clade, we found a single ortholog in gymnosperms, *Amborella* and eudicots while monocots have two or more copies, originating from a duplication event in the ancestor of monocots and subsequent duplication in *O. sativa*. It is now known that in *O. sativa* four BBM-like genes have a redundant function in early embryogenesis (Khanday et al., 2018), possibly monocots have retained more copies of BBM because of a selection pressure to maintain a redundant function between all the copies in case a mutation arises that could compromise early development of the plant. But as our phylogenetic reconstruction includes species that belong only to the Poaceae family, an extensive sampling of monocots would be helpful to detect if this retention of copies is exclusive to Poaceae or is a trait common to all monocots. The PLT clade resolved in our phylogenetic reconstruction indicates that a possible duplication of an ancestral PLT1-like gene originated the PLT5 and PLT1-2/PLT3-7 clades in angiosperms. None of the euANT sequences from *A. trichopoda* and *O. sativa* were resolved within the PLT1-2/PLT3-7 clade, this could be due to extinction of the members of this clade or to sampling error. The PLT1-2/PLT3-7 clade has diversified more in eudicots than in other angiosperms and there is evidence that paralogs like PLT1-2 and PLT3-7 have redundant function and similar expression patterns (Horstman et al., 2014). We also recovered a small clade (<50% bootstrap support) of sequences belonging to *S. bicolor, P. abies*, and *S. cucullata* sister to the euANT clade, these sequences have more variable residues in the R1-linker-R2 region with respect to the other sequences in the alignment (see alignment matrix in Supplementary Figure 2), and could be in the way to pseudogenization via the accumulation of mutations after a possible event of duplication (Moore and Purugganan, 2005).

Overall, considering streptophyte algae and previously underrepresented bryophyte, lycophyte, and fern sequences in the reconstruction of ANT phylogeny helps us broadly understand the evolution of this lineage of TFs, as some nodes within the phylogenetic tree are not significantly supported by resampling. Our results suggest that a duplication of a single preANT ancestor gene originated the land plant-exclusive basalANT and euANT lineages and support that, as Floyd and Bowman (2007) hypothesized, the most recent common ancestor of embryophytes had one basalANT and one euANT protein encoded in its genome. It would be interesting if this hypothesis holds and the relationships between ANT gene clades is resolved with greater support as new genomes of streptophyte algae and non-seed plants are published and added to the phylogenetic analysis of ANT TFs.

### ANT Protein Structure Analysis

**FIGURE 2:**
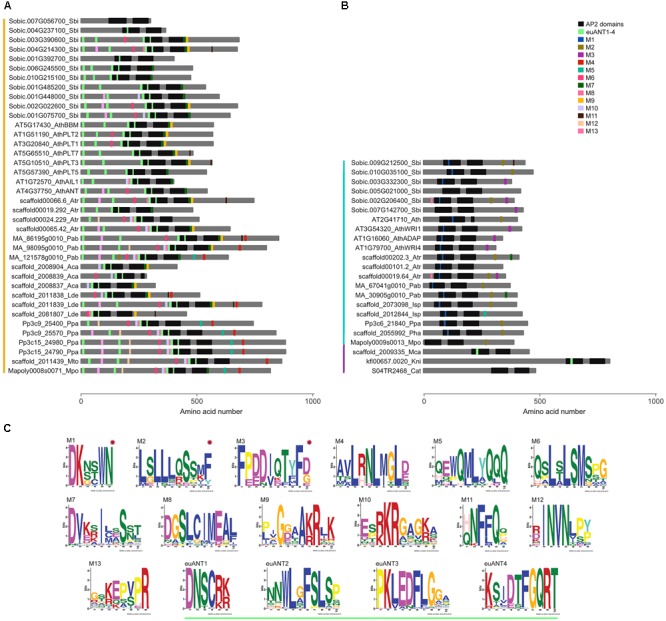
ANT protein conserved motifs in streptophyte sequences. Distribution of motifs identified by MEME in **(A)** euANT, **(B)** basalANT, and **(C)** preANT proteins from representative species of major streptophyte lineages. Each motif (M1 to M13) is represented with a different colored box along a gray line that represents the amino acid (AA) sequence of the protein. Deeply conserved AP2-R1 and AP2-R2 domains are shown as black rectangles of approximately 76 AAs. Motifs euANT1- 4, also identified in this study and reported previously by Kim et al. (2006), are displayed with light green boxes. Colored vertical lines left to taxa denote euANT AA sequences in orange, basalANT in cyan and preANT in purple. **(C)** Logo of each motif identified by MEME. basalANT-exclusive motifs are marked with a red star. See Supplementary Figure 3 for extended MEME results of all the ANT sequences in this study.

In order to characterize ANT protein structure among streptophyte organisms, and to identify putative differences that could distinguish groups formed in our phylogenetic analysis, we first focused in analyzing the position and AA composition in AP2-R1 and AP2-R2 domains, based on the previously description of such domains (Riechmann and Meyerowitz, 1998; Kim et al., 2006). For this we used the retrieved sequences (enlisted in Supplementary Table 1) and generated a *de novo* motif search using MEME^4^ (Bailey and Elkan, 1994). In the sequences analyzed, euANT proteins range from 263 to 882 AAs in length (Figure 2A), while those of basalANT from 309 to 472 AAs (Figure 2B). In the proteins we denominated as preANT, lengths are 436, 455, and 803 AAs for *C. atmophyticus, M. caldariorum*, and *K. nitens*, respectively (Figure 2B). All euANT and preANT AA sequences have a long pre-domain region while all basalANT proteins possess a short pre-domain region. This could mean that the ancestral streptophyte ANT protein likely had a long pre-domain region that was lost in the basalANT lineage, contrasting with Kim et al. (2006) that hypothesized that a short pre-domain region was the ancestral state of ANT proteins. Such conclusion may be influenced by the fact that basalANT-like proteins with a short pre-domain were recovered as sister sequences to the basalANT and euANT clades in their phylogenetic reconstruction of ANT while our new phylogenetic reconstruction, which includes sequences from algae, suggests a loss of the long pre-domain region in the basalANT clade.

The respective lengths of AP2-R1 and AP2-R2 domains are highly conserved in the majority of the AA sequences analyzed in this study. The AP2-R1 domain ranges from 72 to 77 AAs and the AP2-R2 domain from 65 to 70 AAs. Some exceptions are the euANT sequence AT5G10510_AthPLT3 that has an AP2-R1 motif of 98 AAs, and AT2G41710_Ath, Solyc08g076380_Sly, and Os05g45954_Osa all basalANT sequences with expanded AP2-R1 domains of 110, 83, and 89 AAs (Supplementary Figure 2). Sequences with a reduced version of the AP2-R1 domain include just euANT sequences from eudicots; Solyc03g123430_Sly, with 64 AAs; Os07g03250_Osa, with 33 AAs, and Os03g07940_Osa with 38 AAs. The fern euANT sequences scaffold_2008839_Aca and scaffold_2027582_Phe possess a reduced version of the AP2-R2 domain, with 17 and 24 AAs, respectively. In the same manner, the *Amborella* sequence scaffold00024.229_Atr has an AP2-R2 domain of 54 AAs and the eudicot sequence Solyc03g123430_Sly, of 60 AAs. Two basalANT sequences also have a different AP2-R2 domain length wise, AT2G41710_Ath and Solyc08g076380_Sly with 15 and 102 AAs, respectively. The length of the AP2 domains in preANT proteins is within the range estimated for the majority of the sequences in this study. It would be interesting to test if ANT proteins with reduced AP2 domains can still bind DNA and, if so, what is the sequence of the *cis*-element to which they bind. We also found that the linker region is deeply conserved between all the sequences analyzed (see also alignment in Supplementary Figure 2). It is possible that changes in this region resulted deleterious, this suggests its importance in correct protein structure and function.

Some of the most conserved motifs found by MEME reside within the AP2-R1 – linker – AP2-R2 region of ANT proteins. The euANT1-4 motifs, identified by Kim et al. (2006), were represented in our MEME results (light green boxes in Figure 2) in sequences belonging to the euANT clade. One of the euANT sequences from *S. bicolor*, Sobic.007G056700, has a divergent 10-AA insertion in the AP2-R1 domain and therefore does not have the euANT1 motif. Moreover, MEME did not find any other of the euANT motifs in this protein. This divergence is only present in some of the monocot sequences analyzed, as another two sequences from *O. sativa*, Os03g07940 and Os07g03250 appear to have lost all euANT motifs. We found that 69% of euANT sequences have the euANT2 motif, 79% the euANT3 motif and 90% the euANT4 motif (Figure 2). A previous euANT lineage characterization stated that all euANT proteins possess the four euANT motifs (Kim et al., 2006) but as more sequences from more taxa are considered, we observe that this is not the rule for all plant lineages.

Other conserved motifs shared by ANT proteins besides euANT1-4 were named M1 through M13. Motifs shared by basalANT and euANT proteins are M5, M7, M11, and M13. The M5 motif (Figure 2A,B, cyan boxes) is located downstream of the AP2-R2 domain in basal and euANT sequences. In euANT proteins, M5 is only present in bryophytes and gymnosperms, suggesting that it was lost in ferns and in the lineage that gave rise to angiosperms. M7 motif, present in 53% of the sequences analyzed, is located downstream of the AP2-R2 domain of euANT genes and its sequence suggests it could be a nuclear localization signal (Aida et al., 2004). The M11 motif (Figure 2A,B, brown boxes) in the euANT clade is present in lycophytes, gymnosperms, and angiosperms and is absent from bryophytes and ferns, suggesting it appeared in the ancestor of tracheophytes. Only monocot sequences from the basalANT clade share M11, this may be product of a convergent evolution of this motif in both clades or of a loss of this motif in the euANT sequences from ferns and basalANT sequences from all lineages except monocots. Also, the M13 motif (Figure 2A,B, pink boxes) could have arisen independently in both clades, as euANT proteins belonging to the AIL1/ANT clade (Figure 1) and monocot proteins from the BBM clade share it, while only six basalANT proteins from angiosperm species have the M13 motif (Supplementary Figure 3).

Motifs exclusive to the basalANT clade of proteins are M1, located within the 10-AA insertion in the AP2-R1 domain (Figure 2C, red asterisks); the Leucine-rich M2, situated toward the C-terminus of the protein and shared by 54% of the sequences and M3. M3 is shared by angiosperm sequences of the WRI/ADAP clades (Figure 1). These sequences are the only members of basalANT with known function in fatty acid metabolism and abscisic acid response. It would be interesting to test if the acquisition of the M3 motif in angiosperms has a functional benefit, influencing protein 3D structure, binding to DNA or interaction with other proteins to regulate transcription of target genes.

euANT clade-exclusive motifs are M4, M6, M8, M10, and M12 (Figure 2C). M4 is a motif located toward the C-terminal side of the protein and is absent from angiosperm sequences (Figure 2A, red boxes). M6 is present in sequences from bryophytes, lycophytes, ferns, and gymnosperms, as well as in angiosperm protein sequences that belong to the AIL1/ANT, BBM, and PLT1/PLT2 clades (Figure 1, 2A, in magenta), this suggests M6 was lost before the PLT3/PLT7 – PLT5 divergence. El Ouakfaoui et al. (2010) reported this same motif (M6) in vascular plant sequences and named it euANT5. The M8 motif (Figure 2A) follows the same tendency, with the exception of PLT1/PLT2-type sequences which lacks it. Conserved motifs M10 and M12 (Figure 2A, in light purple and peach boxes) are present in bryophyte, gymnosperm and angiosperm sequences from the AIL1/ANT and BBM clades (Figure 1). We found that the M12 motif was absent from lycophytes and ferns. Our MEME search detected a single conserved motif within the *K. nitens* and *M. caldariorum* sequences, the euANT1 motif. The presence of euANT1 and the length of the pre-domain region in preANT proteins (Figure 2B) suggests that the ancestral ANT sequence resembled more an euANT protein and that subsequent changes in the structure of ANT protein, including the length of the pre-domain region and AP2-R1 domain sequence gave rise to the basalANT lineage of proteins. euANT members from bryophytes possess almost all the euANT conserved motifs found in our MEME analysis, this suggests that the most recent common ancestor of embryophytes already beared these motifs. If the conserved motifs found have a functional role, the loss of motifs in certain angiosperm euANT sequences suggests that subfunctionalization took place after gene duplication and divergence in this lineage.

Whether the differences in motifs among the ANT-like proteins explored in this study, both those first described here and those previously described elsewhere, are determinant for the function of these TFs remains to be explored *in planta* by using the few plant model systems available so far.

### Selection Analysis for ANT Genes

The adaptation of plants to a desiccated environment is, in high degree, the result of whole genome duplications and subsequent changes in coding, non-coding and regulatory sequences that augment the diversification potential in plant lineages and that selection will act upon (Van de Peer et al., 2009; Wittkopp and Kalay, 2012). To find evidence of the type and strength of natural selection acting along ANT gene evolution, we performed two types of FEL site-wise selection analyses using the ratio of non-synonymous to synonymous substitutions (dN/dS) along the codon alignment of the euANT and basalANT coding sequences (Kosakovsky Pond and Frost, 2005). When the euANT branches were selected as foreground and basalANT branches as background, the dN/dS value (*w*), which indicates the ratio of non-synonymous to synonymous substitutions, was 0.3781 for euANT and 0.2720 for basalANT. Similar *w* values were obtained when basalANT branches were selected as foreground and euANT as background, 0.2704 and 0.3782, respectively. This suggests that the basalANT and euANT clades mainly underwent negative or purifying selection (*w* < 1), which could explain the relatively low sequence variability in the AP2R1-linker-AP2R2 region that binds to DNA and allows transcriptional regulation of ANT target genes. This analysis also suggests that basalANT genes have been through stronger purifying selection than euANT genes (Supplementary Table 2), which have accumulated more changes in the pre- and post-domain regions after tracheophyte divergence.

The site-specific selection analyses carried out along the 2,612 codons in the ANT genes alignment, revealed that 17 sites are under positive selection and 302 sites are under negative selection constantly along the ANT phylogeny (with a *p*-value < 0.05 for the test dN≠dS) in the euANT clade as calculated by FEL. While the basalANT clade coding sequences have 6 and 235 sites under constant positive and negative selection pressure, respectively (*p* < 0.05). All the euANT and basalANT sites that are unusually variable and are under positive diversifying selection, fall outside the region that codes for the two AP2 domains and the linker region of the protein (Supplementary Figure 4B). The MEME analysis supported seven of the sites detected by FEL, as having positive selection in the euANT clade and two of the sites in the basalANT clade (*p* < 0.05). MEME test detected that another 34 sites are under episodic diversifying selection (*p* < 0.05, Supplementary Table 2). These results are evidence that the majority of the coding sequence changes, occurring in the pre- and post-domain regions of both basalANT and euANT through time, might have been caused by neutral mutations that did not affect the fitness of the proteins.

Finally, because it has been shown that certain ANT genes respond to the WUSCHEL-related homeobox 5 (WOX5) TF (Ding and Friml, 2010) and to phytohormones like auxins (Aida et al., 2004; Li and Xue, 2011; Aoyama et al., 2012; Yamaguchi et al., 2016), cytokinins (Dello Ioio et al., 2008; Li and Xue, 2011), and ethylene (Li and Xue, 2011), we made an exploratory computational exercise to identify the putative association of diverse transcriptional regulators with the regulatory regions of ANT genes among Streptophyta. For this, we searched for ERF, ARF, ARR-B, and WOX/WUSCHEL-responsive *cis-*elements found in the promoter of ANT genes from *O. sativa, A. thaliana, A. trichopoda, P. patens, M. polymorpha, K. nitens*, and *C. reinhardtii*.

On average, auxin response elements (AREs) are the most abundant motifs in the ANT regulatory regions analyzed (Supplementary Figure 4A and Supplementary Table 3). euANT genes regulate embryogenesis, stem cell niche maintenance and organ growth both in the shoot and root of *A. thaliana* by their interaction with the phytohormone auxin (Horstman et al., 2014). Also, auxin-induced euANT genes from the moss *P. patens* act as molecular switches for the development of different types of stem cells and three-dimensional growth (Aoyama et al., 2012). Interestingly, it has been reported that the auxin perception pathway composed by TIR1-Aux/IAA-ARF is not present in *K. nitens* (Ohtaka et al., 2017), however, we found four AREs in the promoter region of the preANT gene of this species. In light of these findings, our results suggest that, although AREs are present in the promoter regions of algae ANT genes, their putative ARF-mediated interaction with Auxins was acquired later in the common ancestor of embryophytes and could have allowed the development of 3D apical growth (an innovation in the transition to land), regulation of stem cell identity, and organ size control (Harrison, 2017). The second most abundant CRE is the WOX/WUSCHEL TF family DNA-binding motif (Supplementary Figure 4). All the sequences analyzed contain at least one WOX/WUSCHEL-responsive motif, being potential targets of such TFs, except for the promoter region of the basalANT gene *Mapoly0009s0013* (Supplementary Table 3). It has been demonstrated that WOX5 acts downstream of Auxin to regulate PLT1 expression in the root apical meristem of *A. thaliana* (Ding and Friml, 2010). Green algae and basal plant lineages contain WOX genes belonging to the ancient clade of WOX TFs and possess the conserved homeodomain motif that binds to DNA to regulate transcription of target genes (Lian et al., 2014). From our *in silico* exploration, we hypothesize that a WOX-ANT genetic interaction could have arisen in the common ancestor of streptophytes. We speculate that this ancient interaction, in which a WOX TF acts upstream regulating the expression of an ANT gene, may have been conserved in modern plants. A possible example of this is the WOX5- *PLT1* genetic interaction, two major factors for root stem cell niche maintenance in *A. thaliana* (Aida et al., 2004). Potential transcriptional regulation of ANT genes in response to cytokinin can also be inferred by the presence of ARR-B CREs in promoter sequences representative of every major streptophyte lineage (Supplementary Table 3). Holzinger and Becker (2015) found that orthologs of the cytokinin signaling in the streptophyte algae *Klebsormidium cernulatum* were up-regulated upon desiccation. Although the interaction between cytokinin and ANT genes could have been originated in the common ancestor of streptophytes, it would be interesting to test experimentally if the angiosperm basalANT genes *WRI1, WR14*, and *ADAP* are direct transcriptional targets of ARR-B because of their role in adaptation to desiccation. We also found that on average, ERF-responsive CREs (named GCC boxes; Fujimoto et al., 2000) were the least common *cis*-elements in *ANT* genes from streptophytes. Only four basalANT promoters had GCC boxes and corresponded to angiosperm sequences (Supplementary Table 3), this suggests that a putative interaction between ERFs and basalANT genes was acquired after the duplication of this genes in angiosperms.

Our results on the presence and abundance of these CREs in the promoter regions of the analyzed pre, basal and euANT genes, are only predictive for those genes and species. So far, there is no experimental evidence that indicates they do respond to a given hormone or that TFs indeed bind to them. To demonstrate that a specific hormone influences, through the binding of a given TF, gene transcription future wet-lab experiments are needed.

In summary, here we explored and analyzed in a more complete and deep manner the putative origin of basalANT and euANT TFs. This is the first study where streptophyte algae sequences have been identified and analyzed for these TFs. Moreover, our approach also explores differences in protein motifs and *cis*-regulatory elements in the regulatory sequences of representative genes along the plant tree of life. Finally, our natural selection analysis reveals those sequences of the genes that have been subject to changes versus those which remain highly conserved. Altogether, our results provide a broad framework for ANT-like genes putative functions in the evolution of plant development, which we assume is useful for the generation of novel hypotheses about protein motif importance and *cis*-elements relevance in ANT gene expression that could be validated experimentally, in order to define the relevance of ANT TFs in the conquest of land by plants.

## Author Contributions

MD-Á and AC-R conceived the study and wrote the manuscript. MD-Á performed analyses.

## Conflict of Interest Statement

The authors declare that the research was conducted in the absence of any commercial or financial relationships that could be construed as a potential conflict of interest.

## Footnotes

1 https://phytozome.jgi.doe.gov/pz/portal.html

2 ConGenIE.org

3 https://db.cngb.org/onekp/

4 http://www.plantmorphogenesis.bio.titech.ac.jp/~algae_genome_project/klebsormidium/

5 http://meme-suite.org/

6 https://phytozome.jgi.doe.gov/pz/portal.html

7 https://www.ncbi.nlm.nih.gov/
