# Supplementary material for: A Phylogenetic Study of the ANT Family Points to a preANT Gene as the Ancestor of Basal and euANT Transcription Factors in Land Plants
Source: Front Plant Sci. 2019 Jan 29;10:17. doi: 10.3389/fpls.2019.00017 (PMC6361745; doi:10.3389/fpls.2019.00017)
Supplement: Supplementary file 4 [file Image_1.pdf]

Supplementary Material

A Phylogenetic Study of the ANT Family Points to a preANT Gene as the Ancestor of Basal and euANT Transcription Factors in Land Plants

Melissa Dipp-Álvarez, Alfredo Cruz-Ramírez*

*** Correspondence:**

Alfredo Cruz-Ramírez: alfredo.cruz@cinvestav.mx

1 Supplementary Figures and Tables

1.1 Supplementary Figures

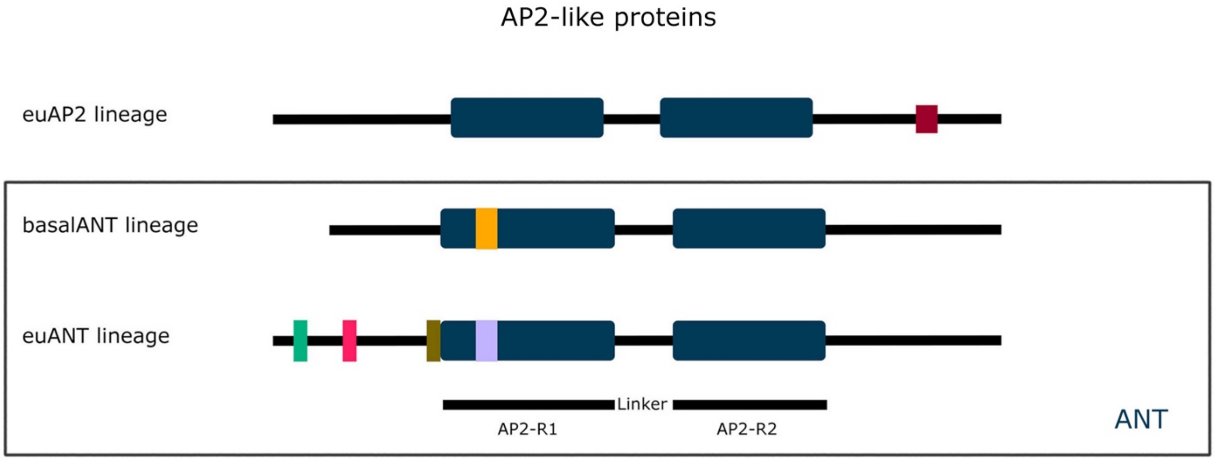

Supplementary Figure 1. Structure of *AP2-like* proteins. ANT lineage proteins are outlined. Blue boxes indicate AP2 domains. Light purple, brown, pink, and green boxes indicate euANT-specific motifs. Yellow region in basalANT AP2-R1 domain shows portion of the 10-amino acid insertion that is not conserved. Red box in euAP2 lineage shows site of the post domain region where a miR172 binding motif is located (Kim *et* al., 2006).

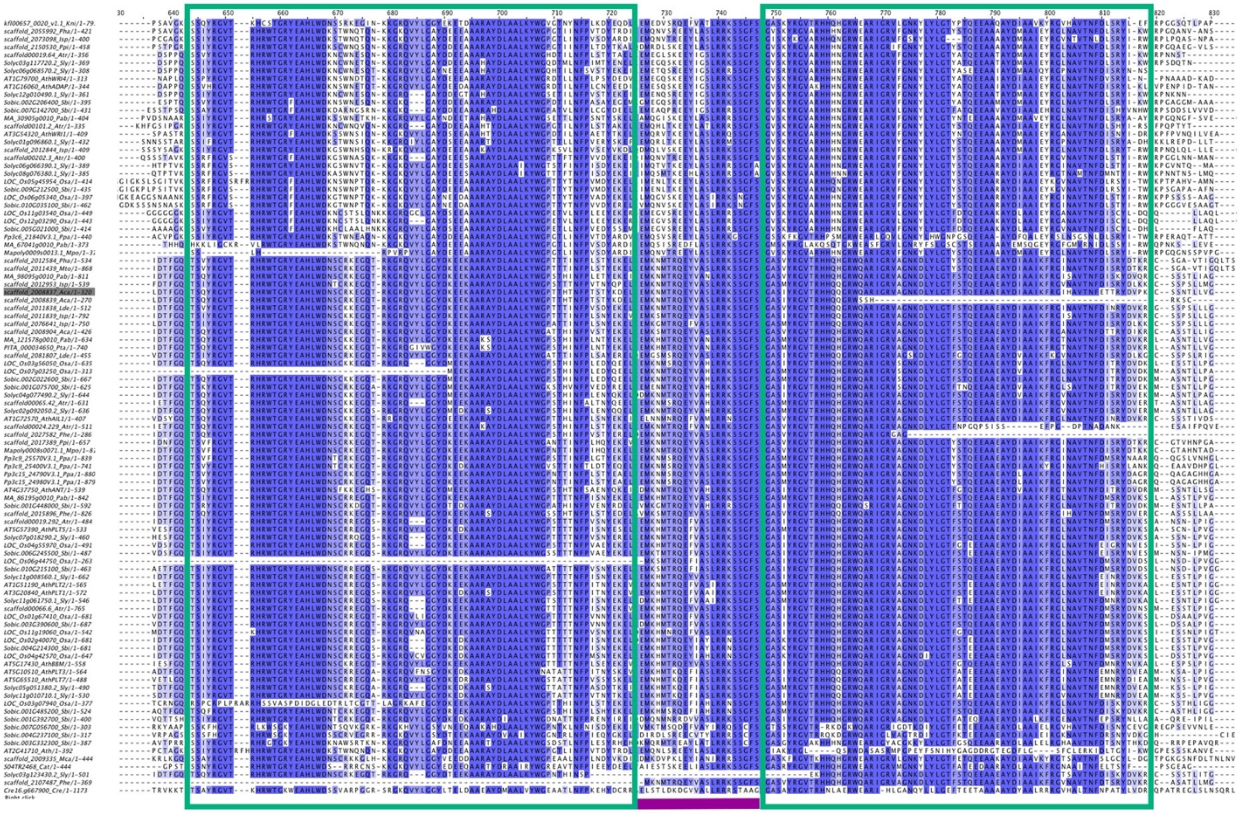

Supplementary Figure 2. AP2-R1, linker, and AP2-R2 region of the amino acid sequences alignment made with MAFFT (Katoh et al., 2017). Green rectangles mark AP2 domains. Purple line indicates linker region.

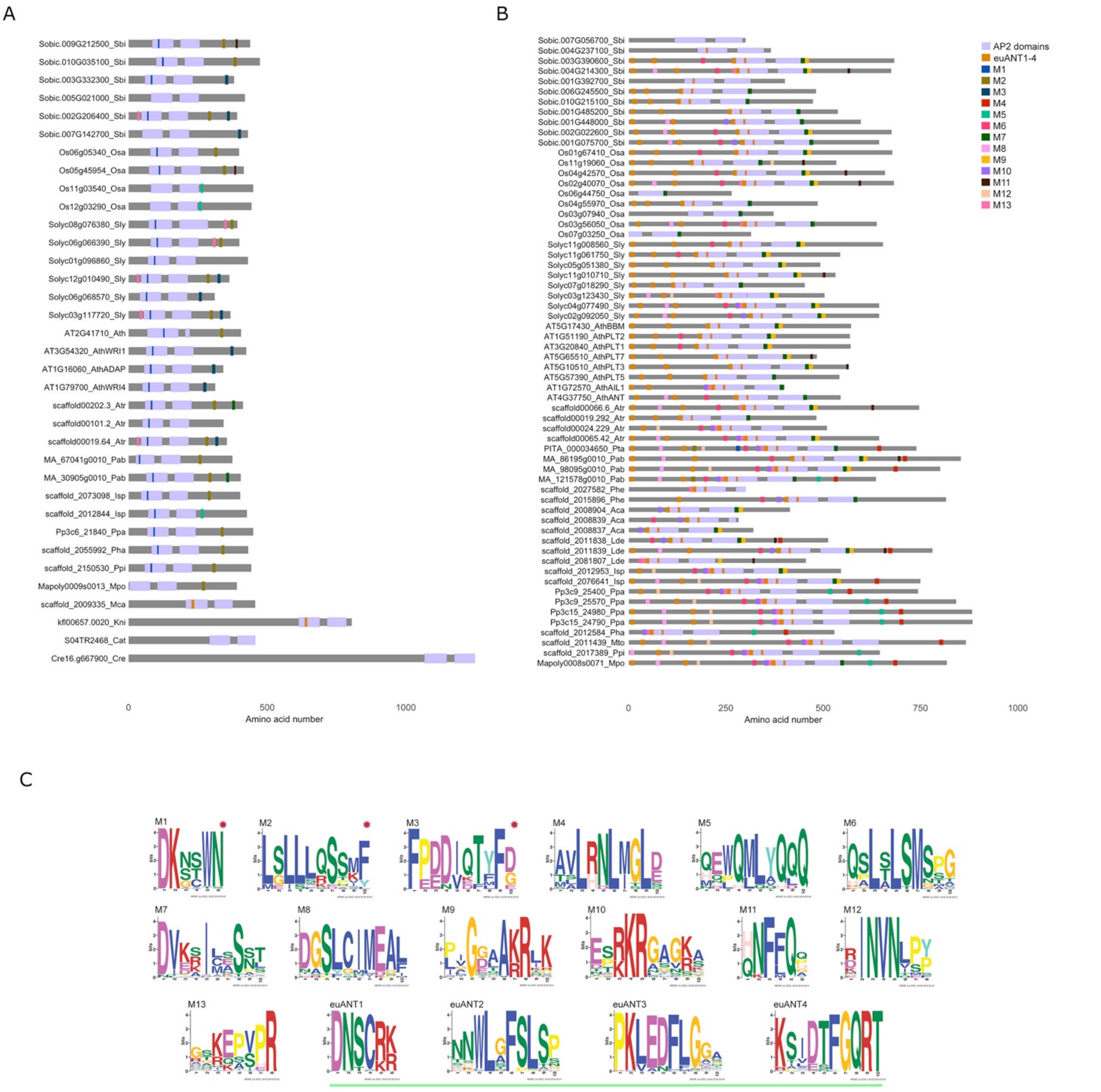

Supplementary Figure 3. Distribution of motifs identified by MEME in **(A)** basalANT and preANT and **(B)** euANT proteins retrieved in this study. Each motif (M1 to M13) is represented with a different colored box along a gray line that represents the amino acid (AA) sequence of the protein. Deeply conserved AP2-R1 and AP2-R2 domains are shown as light purple rectangles of approximately 76 AAs. Motifs euANT1- 4, also identified in this study and reported previously by Kim et al. (2006), are displayed with orange boxes. **(C)** Logo of each motif identified by MEME. basalANT-exclusive motifs are marked with a red star. Light green line denotes euANT1-4 motif logos.

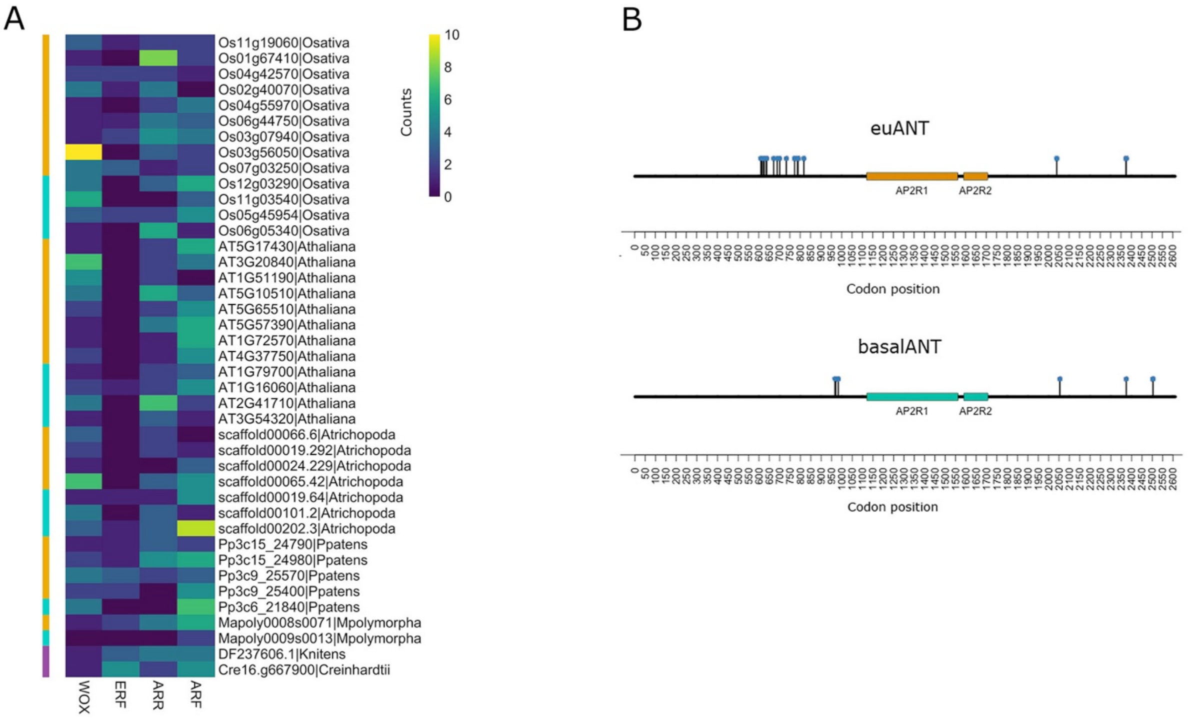

Supplementary Figure 4. Cis-regulatory elements (CREs) in streptophyte ANT gene promoters and positive selection in ANT protein coding sequences. **(A)** Total counts of WUSCHEL-related homeobox (WOX), ethylene response factor (ERF), auxin response factor (ARF) and cytokinin response regulator (ARR-B) cis elements in each ANT gene promoter region. Bar left to the heatmap indicates euANT genes in orange, basalANT genes in turquoise and algae genes in purple. **(B)** euANT and basalANT codons under positive diversifying selection detected by FEL analysis. Black line represents ANT protein coding sequence. Regions coding for AP2 domains are mapped along the line as orange and turquoise boxes for euANT and basalANT, respectively. Sites with significant evidence of positive selection ($p < .05$ for the test $dN \neq dS$) are displayed with blue tags along the line.
